# Quantification of corruption in preventative cost-based S-LCA: a contribution to the Oiconomy project

**DOI:** 10.1007/s11367-018-1507-z

**Published:** 2018-09-06

**Authors:** Pim R. Croes, Walter J. V. Vermeulen

**Affiliations:** Copernicus Institute of Sustainable Development, Princetonlaan 8a, 3584 CB Utrecht, The Netherlands

**Keywords:** Corruption, ESCU, Social LCA, Profit margin, Oiconomy standard, Preventative costs, Sustainability

## Abstract

**Purpose:**

Corruption is one of the key social aspects, heavily impacting all three Planet-, People- and Prosperity sustainability pillars and is therefore essential to be included in S-LCA. The objective of this article is to consider the available options to quantify corruption in preventative cost-based S-LCA, and to make a first proposal for quantification.

**Methods:**

Literature was investigated on potential S-LCA assessment methods of corruption. To date, such literature is hardly available, so more generally, S-LCA methods were assessed on described concepts and ideas, and assessed on five criteria. Following this, using the obtained conclusions and ideas, a proposal for the quantification of corruption for the preventative cost-based Oiconomy system was developed, following the five-step Oiconomy method (Croes and Vermeulen in J Clean Prod 102:178–187, 2015).

**Results and discussion:**

Based on some examples, Dreyer et al. (Int J Life Cycle Assess 15(3):247–259, 2010) argue that various social aspects, including corruption, are better assessed by companies’ preventative efforts than by their impact. Therefore, modifying a method developed by Dreyer et al. (Int J Life Cycle Assess 15(3):247–259, 2010), an indicator is proposed provided by the product of the marginal preventative costs and the quality of a companies’ preventative governance. For the aspect of corruption, the internationally accepted target is “zero tolerance.” Literature shows that the ultimate business choice under pressure of corruption is “not doing the business.” Because profitability is the main driver for companies, refraining from the business is proposed as the marginal preventative measure, and the related profit as the maximum quantitative indicator for S-LCA. For the risk factor, a technique is proposed based on scoring a company’s governance quality by checking the four Plan-Do-Check-Act effort classes of common risk-based certification standards’ criteria.

**Conclusions:**

Our assessment shows a definite need for the inclusion of the aspect of corruption in S-LCA, but no options for a reasonably certain assessment are available for the aspect of corruption in impact-based S-LCA, also suitable for the preventative cost-based Oiconomy system. However, based on literature-derived ideas and principles, for the Oiconomy system, we could propose both a performance reference point and marginal preventative costs as a quantitative measure for corruption. The proposed measure is not paying the bribe, but the proposed indicator is a governance quality-dependent fraction of the consequentially lost profit margin. Consequences, limitations and possible objections to our proposed methods are discussed.

**Electronic supplementary material:**

The online version of this article (10.1007/s11367-018-1507-z) contains supplementary material, which is available to authorized users.

## Introduction

The definition of corruption by the United Nations Global Compact, Transparency International and the World Bank is: “The abuse of entrusted power for private gain” (United Nations Global Compact and Transparency International 2009; World Bank 2008, p.16), including bribery of public officials, embezzlement, trading in influence, abuse of function, illicit enrichment by public officials, money laundering, and obstruction of justice (United Nations Global Compact 2010, p.13). But tax evasion, support of politicians or political parties by businesses, and even lobbying are also regularly perceived as corruption (OECD 2013).

Corruption, defined as a crime by the 2004 UN Convention against Corruption, signed by 140 countries and legally implemented by many countries, extends the concept of private gain to also “another person or entity,” this way including the various types of favoritism (United Nations Office on Drugs and Crime 2004, article 15), such as nepotism, clientelism and cronyism. Although an OECD convention regulates information transfer on taxes between countries (OECD and The Council of Europe 2012), there is no international agreement on aspects like tax evasion and political involvement. Because company-specific data on their involvement in bribery- and favoritism-related activities usually are illicit, but data on their paid taxes available, quantification of these aspects requires different methods. Therefore, for the purpose of this paper we choose to define “corruption” as all bribery- and favoritism-related activities, and exclude the aspect of tax evasion.

On December 17, 2010, the Tunisian graduate Mohamed Bouazizi, previously bullied by the police and desperate because he had insufficient money to bribe the police for allowing him to sell produce from his unlicensed vegetable cart, set himself on fire (BBC News 2011). The subsequent protests grew to revolt and war in the Arabic world, causing hundreds of thousands of deaths, millions of wounded and displaced people and religion- and migration-based fear and hatred, infecting greater parts of the world. Although the underlying causes are multiple, this is a clear example of the huge impact that corruption may have. It also illustrates that measuring or predicting the impact of a social stressor with some certainty is a very challenging task.

A large body of literature demonstrates the detrimental impact of corruption. The World Bank’s website calls corruption the single greatest obstacle to economic and social development (World Bank 2016), and various authors point to the fact that corruption shifts governments spending away from health, education and infrastructure maintenance, e.g. (Campos and Pradhan 2007; International Chamber of Commerce et al. 2008; Mauro 1997; Wei 1999; Søreide 2014). Transparency International states that there is clear evidence that corruption is one of the primary causes of Millennium Development Goals being off-track (Fagan et al. 2010). Corruption has a detrimental influence in all three sustainability pillars, which are often referred to as the triple bottom line “PPP”: Planet, People, Profit (Elkington 2004), but more recently as Planet, People and Prosperity, stressing the structural causes of inequality and poverty, and also explicitly addressed in the UN’s Sustainable Development Goals (European Commission 2002; Barkemeyer et al. 2014; Vermeulen and Witjes 2016; SDSN 2015; Gupta and Vegelin 2016).

Considering the planet category, the first striking fact is that many of the major oil producing countries score as very corrupt in the Corruption Perceptions ranking, e.g., China (77), Mexico (135), Russia (135), Iran (130), Nigeria (148), Venezuela (169), Iraq (169), Libya (171) (Transparency International 2017). But the oil business is just an example of the mining industry, which, according to Transparency International, is one of the business sectors most likely to be sensitive to bribery. Because green industry also heavily depends on mining, corruption may be expected. Gallium for photovoltaic cells, tantalum for microelectronics, rear-earth metals for magnets in windmills, platinum for catalysts and many other mined resources used in green industry, are sourced in countries with very high corruption perceptions ranking scores (Transparency International 2013, p.199). For both climate and biodiversity, loss of forests is one of the major threats. According to various authors, corruption is one of the root causes of forest degradation. (Hicks 2013; Koyunen and Yilmaz 2009; Transparency International 2013; FAO 2001).

Considering the people category, corruption plays a key role in health and social wellbeing. In the first place it has an impact on poverty, as demonstrated by Gupta et al. (2002). In 1996, the then-president of the World Bank, James D. Wolfensohn, declared: “For developing countries to achieve growth and poverty reduction, we need to deal with the cancer of corruption” (Bhargava 2006, p. 1). Corruption especially affects health, because in development countries it prevails in water and sanitation management and in the health sector itself, in this way representing a direct death toll, especially of children (Azfar and Gurgur 2008; Hanf et al. 2011; Factor and Kang 2015).

We also find a corruption-related death toll in natural disasters, because of its impact from supposedly disaster-proof constructions, causing landslides and collapsing dikes and buildings. And finally, in development countries corruption affects education, e.g. (Azfar and Gurgur 2008; Hallak and Poisson 2005).

Considering the prosperity category, estimates show that the cost of corruption equals more than 5% of global GDP (US $2.6 trillion), with over US $1 trillion paid in bribes each year. Corruption adds up to 10% to the total cost of doing business globally, and up to 25% to the cost of procurement contracts in developing countries” (International Chamber of Commerce et al. 2008, p. 1). “Corruption is shown to have a significant negative effect on entrepreneurship, and businesses see themselves as victims of corruption” (Runde et al. 2014, p. 1). Bribery and taxation are closely related. By corruption, tax systems are biased (Gupta et al. 2002, p. 25), and low taxes in “tax paradises” impact taxation in the countries of production. However there is a body of literature indicating that in some cases corruption “greases the wheels” of economic growth, especially under conditions of low quality governmental institutions and low economic freedom, the effect of which however, decreases or even reverses at improving institutions and economic freedom, e.g. Heckelman and Powell (2008, p. 17).

### LCA literature on corruption

The UNEP guidelines for Social Life Cycle Assessment (S-LCA) of products mention corruption as a subcategory (UNEP/SETAC 2009, p. 49). But to date, the literature on addressing corruption in LCA is very limited. Some authors, usually citing the guidelines, mention corruption as an aspect to be studied, e.g. (Grießhammer et al. (2006), Benoît et al. (2010). Dreyer et al. (2010), describing a method for labor conditions, mentions its applicability for corruption. Ekener-Petersen and Finnveden (2013) identify corruption as a potential hotspot in the life cycle of a laptop computer. No attempt at further quantification of the specific aspect of corruption or research into potential indicators for that purpose could be found in the literature. We may conclude that there is a research gap in the quantification of corruption in S-LCA.

## Objective

This paper is a contribution to the Oiconomy project, comprising the development of a LCA system based on the costs of prevention instead of the assessment of the damage itself. The Oiconomy system comprises both environmental, social, and economic sustainability aspects and intends to use a certification system for the transfer of foreground data through the supply chain of products. The system is fully explained in Croes and Vermeulen (2015) and will be described shortly in a subsection “Oiconomy system” in the results section of this paper, where it is presented as part of a study of S-LCA methodology. The objective of this paper is to investigate literature on the available options to (preferably interval-) quantify corruption in S-LCA, distinctive for specific products and use the options and ideas found to make a first proposal for quantification for preventative costs based S-LCA.

## Methods

As a first step, literature was investigated on potential S-LCA assessment methods of corruption. Combining the words “corruption,” with “S-LCA,” “Social LCA” or “life cycle assessment,” with Scopus 3 articles were found and with Google Scholar 256 articles. However, none of these dealt with quantification of corruption. Also the words “bribery” and “tax evasion” instead of corruption did not result in relevant articles. Therefore, using literature reviews, their leads and references of scholars in the field, the 12 most used of which are listed in the results section, S-LCA methods were assessed more generally on described concepts and ideas, and assessed on the following five criteria:

1. The method should give a (preferably interval) quantitative, objective and certain measure.
2. The required data must be available and data collection preferably feasible for industry.
3. The measure must be distinctive for a specific product or company.
4. The results must be aggregable with those for other environmental and social-economic aspects.
5. The method must be suitable to be used for the aspect of corruption.

These 5 criteria were chosen in order to assess the feasibility of the investigated S-LCA methods for the quantification of the social sustainability, including corruption, of specific products, generically applicable for different sustainability aspects and different product categories.

Subsequently, using the obtained conclusions and ideas, a proposal for the quantification of corruption for the preventative cost-based Oiconomy system was developed, following the Oiconomy method (Croes and Vermeulen 2015), which is very similar to the method used in the regularly used EcoCost system (Vogtländer et al. 2000) and consists of five steps:

Step 1. Definition of the impact category and characterization factor.
Step 2. Determination of the corruption target, or performance reference point.
Step 3. Literature study of available preventative measures.
Step 4. Determination of the costs of the available preventative measures.
Step 5. Assessment of which preventative measures are required to globally reach the target. The last and most expensive determined this way, presents the marginal preventative costs, which in the EcoCost and Oiconomy systems serve as cost distance to target, and represent our proposal for the quantification of corruption.

## Results—literature review

The UNEP guideline on LCA only mentions corruption as a subcategory under the stakeholder category of “society” (UNEP/SETAC 2009, p. 49). The Global Reporting Initiative (GRI) dedicate a more quantitative guideline on corruption, requiring members to report concrete numbers of operations assessed and of risks identified, numbers and percentages of internal people and external organizations to whom the company has communicated its policy, numbers of internal and external people who have received training on anti-corruption, and also to report the number of confirmed incidents (GRI 2013, p. 204–209).

As mentioned in the introduction, no literature could be found on the quantification of the specific aspect of corruption, or research into potential indicators for that purpose. Therefore, we will continue investigating S-LCA methodology in general, on which several literature reviews have been written, e.g. (Jørgensen et al. 2008; Macombe et al. 2013; Benoît and Vickery-Niederman 2011; Chhipi-Shrestha et al. 2015; Swarr 2009; Hsu et al. 2013; Hauschild et al. 2008; Benoît Norris 2012; Hutchins and Sutherland 2008; Jørgensen et al. 2010b; Finkbeiner et al. 2010). Not included in these reviews is preventative cost-based LCA (Vogtländer et al. 2000), extended to S-LCA by Croes and Vermeulen (2015).

Basically, one could assess environmental and socio-economic stressors by their impact, by the measures required to compensate the impact, or by the measures required to prevent them. ISO 14040 defines LCA as: “The compilation and evaluation of the inputs, outputs and the potential environmental *impacts of* a product system throughout its life cycle” (ISO 2006, p. 2). The UNEP guidelines define socio-economic LCA as: “A social *impact* assessment technique that aims to assess the social and socio-economic aspects of products and their potential positive and negative impacts along their life cycle” (UNEP/SETAC 2009, p. 37). Both the ISO standard and the UNEP guidelines intend to increase firms’ and governments’ knowledge and awareness of impacts and provide them with tools to assess the consequences of various alternatives for their products, activities and decisions. Therefore, the main body of LCA and S-LCA literature is impact-based. In impact-based S-LCA (IB-S-LCA) basically two types of methodologies are used, identified by the UNEP guidelines (UNEP/SETAC 2009, p. 71) as Type-I, based on the distance to Performance Reference Points (PRP’s) and categorized in themes of stakeholder’s interests, and Type-II, based on cause-effect pathways.

### Type-I S-LCA assessment methodologies

#### Checklist- and scoring methods

Checklist methods use ticks for a series of indicators usually first belonging to a midpoint impact category and from there to an endpoint category. They assess, for a number of aspects or subcategories, the yes or no presence of impact in the endpoint categories. Scoring methods add a weighting score to the ticks. Franze and Ciroth (2011) compare the social impact of cut roses from Ecuador and from the Netherlands. Based on the number of ticks and the authors’ weighting of the seriousness, a final assessment is made in five grades per aspect. The ticks are based on the authors’ investigation of the involved country and industry sector performance on the area of protection (AoP). Franze and Ciroth apply the method in an indiscriminating way, ticking complete subcategories in order to asses the endpoint of human wellbeing. However, in principle the method can be applied to a midpoint category such as occupational health and safety, or corruption, provided that a balanced set of criteria can be defined as indicators for the impact or status of control.

For further improvement, various authors developed techniques to quantify these dichotomous indicators more precisely, e.g. by means of measuring the opinions of stakeholders and/or experts on the companies’ performance on an indicator. For example, Manik et al. (2013) developed an interval scoring variable for the measurement of the social impact of biodiesel production in Indonesia. By means of questionnaires, for a series of criteria, they determined the distance between a stakeholder’s perceived and expected impact, for each criterion multiplied by an expert panel’s obtained weighting score. A similar technique was used by Foolmaun and Ramjeeawon (2013), studying alternatives for the disposal of PET bottles in Mauritius. They score criteria by the percentage of interviewed stakeholders answering “yes” to questions on their opinion on impact presence. By thereafter transforming these percentages into a five- grade score by equally dividing the 0–100% range into five parts, in principle they introduce a way to aggregate scores for different categories. The authors however point to the fact that all aggregated categories carry equal weight.

Ekener-Petersen and Finnveden (2013), assessing the social impact of the production of a laptop computer, developed a method of scoring a product by a combination of the relative quantity of activities for a product in the different countries along the supply chain, and a rough scoring of the severity of the relevant aspects for the allocation of scores to the product. The activity contribution of countries was determined by the physical weight of the laptop, and by the global producer share of the used resources and manufacturing steps. Weighting was executed by defining hotspots using country performance data on social aspects. However, the authors conclude that data collection is a major issue.

### LCAA

Life Cycle Attribute Assessment (LCAA) was developed by Norris (2006) and further elaborated by Andrews et al. (2009). The technique determines the share of an attribute, such as compliance to a certificate or “child labor-free,” in the supply chain of a product. It is a technique in two steps: 1. Determination of yes or no presence of the attribute and 2: Weighting of the presence by an activity factor, for which Norris takes workhours, as does Hunkeler (2006), but also other factors can be taken (Andrews et al. 2009). The results of LCAA are conclusions such as “the percentage of labor hours that is child labor-free.” However, where Hunkeler only uses country level background data, LCAA is based on both foreground, e.g. by measuring compliance percentages to certificates, and background data, e.g. from Input-Output databases. LCAA uses the simplification of a dichotomous variable per step in a product’s life cycle, inherently normalizes by measuring data as a percentage, and equally weights aspects and steps in the life cycle. Therefore, LCAA gains certainty, especially if combined with certification, but loses accuracy because of lack of gradation. A disadvantage is that the activity factor limits the applicability to aspects that are closely related to the activity factor and therefore also limits the aggregability over different categories.

#### Social hotspot method

Because data on specific companies are lacking, more generic country level data are often proposed as an indicator for the risk of an impact. E.g. the risk of child labor is greater in a country where child labor is common (a “hotspot”), than in a country where it is not common. The disadvantage is of course that these data are very generic and by no means specific for a company or product. Benoit-Norris et al. developed the social hotspot database, which contains both data on social hotspots and on labor intensity for a great number of combinations of countries and sectors (Benoît Norris et al. 2010, 2012). By the inclusion of data on labor intensity per country-specific industry sectors, the determination of worker-related positive or negative effects is facilitated. Although by inclusion of sector data, the granularity of the hotspot method is much improved, it still is far from company specifity. The content of the hotspot database is a strong indicator of the risk of an impact, and useful for risk assessment, but does not really provide data for concrete product- or company assessment before it really has reached company level granularity. That however can only be accomplished by the supply chain actors themselves, combined with a system of independent verification, e.g. certification. Because auditing large numbers of social criteria is expensive, a “risk-based certification system” could be applied, in which frequency, audit time and verified criteria depend on the specific local risk, for which the Hotspot database would be valuable.

Assessing the checklist and scoring methods (CSMs) on our five criteria, we recognize three data sources: Generic country (or region) level data, stakeholders’ and experts’ obtained data, and company obtained data, which sources determine the quality of the assessment.

1. CSMs are quantitative, sometimes even interval quantitative, although usually via a nominal quantitative scoring method. The models based on country-level data are objective and certain if considered as the result for an average product, because contrary to the empirical models (see below) the country-level data used here really refer to the specific country. The data based on stakeholders’ opinions are in the international arena and are fast changing opinions, either biased or very laborious to collect and maintain. Although most of the described CSMs (Ekener-Petersen and Finnveden 2013; Franze and Ciroth 2011; Manik et al. 2013) gave very little granularity, with available foreground data and well-defined sets of criteria these methods can be used for much more detailed assessments, which principle was used by Dreyer et al. (2010) (see below).
2. The data required for CSMs become more difficult to collect, the more certain and specific one wishes them to be. Country-level data-based methods do not discriminate on specific company and product level. The stakeholder-based models are more discriminating, but very laborious and far less certain unless applied on a very limited choice of studied aspects and system boundaries, e.g. used for the aspect of occupational health and safety with the relevant workers interviewed. The hotspot database helps to improve data granularity but will almost by definition never reach company- or product granularity.
3. CSMs are suitable for aggregation within the supply chain for one studied impact category, e.g. employment, but not between categories unless a common relevant normalization denominator can be found applicable to all studied categories.
4. Ignoring the shortcomings on accuracy, certainty and specificity, for the aspect of corruption the country-level data-based models are in principle suitable because country-level data are available from the Transparency International Corruption Perceptions Index (further called “corruption index”). Stakeholder data-based models seem less suitable for corruption, because the stakeholder group is complex, existing of the society and international suppliers and customers, but also of unknown potential business partners who did not take the risk of being involved in corruption.

### Type-II S-LCA assessment methodologies

Chhipi-Shrestha et al. (2015) recognize two types of quantitative Type-II models: 1. The “empirical model” and 2. the “ELCI-database” model. The empirical model constructs an empirical cause-effect pathway based on correlations between established (usually country-level) indicators. The ELCI-database model tries to follow the standard environmental-LCA (ELCA) procedure from life cycle inventory to characterization and sometimes even uses the LCI databases developed for ELCA.

### Empirical type-II models

Considering the empirical model, Norris (2006, p. 99) described a pathway from per capita income to health in the mathematical formula that describes the “Preston curve” relation between income and life expectancy, using World Bank country-level data, but saw too many shortcomings. Feschet et al. (2013) however, argue that, using the Preston curve, and assuming that the specific country location on the curve fully applies to that country, the health increment related to the added value of an activity in a country can be calculated and used as an empirical pathway. They demonstrate that the method works, although only for low income countries, because the Preston curve is almost flat for high income countries. In a similar way, Hutchins and Sutherland (2008) demonstrate a pathway from per capita income to child mortality, enabling a comparison of the effect of added value in different countries to child mortality, and Bocoum et al. (2015) used the relation between the country level GINI (inequality) coefficient and child mortality to develop a pathway from added value to health.

**Fig. 1:**
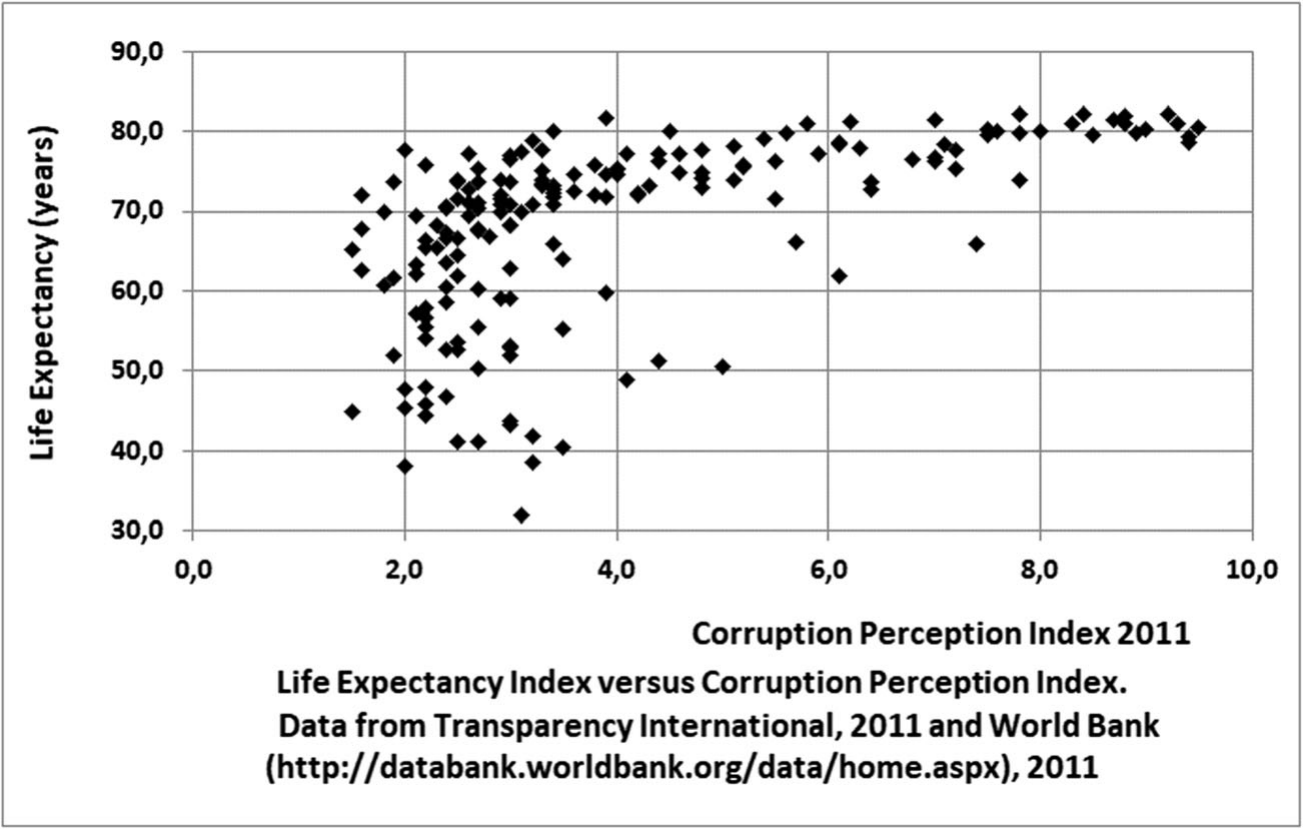
Life Expectancy Index versus Corruption Perception Index. Data from Tranparency International 2011 and World Bank 2011 (http://databank.worldbank.org/data/home.aspx)

**Fig. 2:**
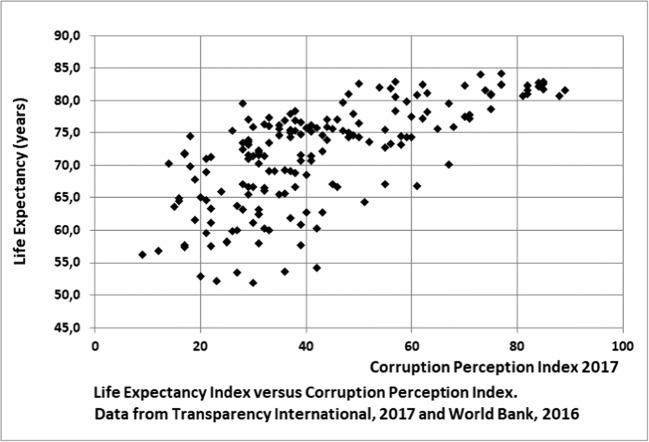
Life Expentancy Index versus Corruption Perception Index. Data from Transparency International 2017 and World Bank 2016

Empirical models are based on a correlation between a single available, but objective, country indicator, such as the GDP or the GINI coefficient, and a S-LCA midpoint category. Most of the authors themselves point out that reality is far more complex, involving a multi-criteria relationship instead. In addition, it is very questionable if the used correlations are causal. For instance, considering the Preston curve, showing the relation between the GDP per capita and life expectancy, there are several other indicators showing a similar relation to life expectancy, for example, relevant for this study, corruption. Figure 1 shows the typical Preston type of relation between the corruption index and the GDP per capita for the year 2011. But Fig. 2, showing a much weaker correlation between the same indicators for the year 2016/17, strengthens our doubt about a causal relationship between corruption and GDP. On the other side, Wu et al. (2015) show, based on multi-criteria statistical techniques, that although there is no significant direct causal relation between income and health, there is a significant indirect pathway from income, via health expenditures to health. However, they did not study governance in general and in our intuitive opinion, it is very likely that governmental governance in general is the leading indicator, leading to Preston-curve like relations with life expectancy for several of its components. Jørgensen, Lai, & Hauschild argue: “If there is no valid impact pathway, there is no way of telling whether and to what extent the indicators that we apply in S-LCA actually represent damage or benefits to the Area of Protection”. The authors studied the validity of the pathway from child labor to wellbeing (Jørgense, et al. 2010b) and from employment to wellbeing (Jørgensen et al. 2010a) and conclude that too many variables and unknowns affect the pathways to develop a valid pathway.

Although recognizing these difficulties, Weidema (2006) is quite optimistic about finding valid pathways and argues that it is better to develop valid pathways for the less complex categories and use other techniques for the more complex categories. Following the common ELCA technique of using concrete biophysical pathways towards the endpoint category of health, expressed in disability-adjusted life years (DALYs), he proposes quality-adjusted life years (QALYs) as a characterization factor for S-LCA. By extending existing disability and disease classification systems (as a percentage of lost life years) with factors for the various social stressors, all established impact pathways leading to human well-being may be effectively interval quantified.

We will now assess the empirical Type-II models on our five criteria.

1. Empirical models are objective and do provide an interval quantitative indicator, because they are based on country statistics, but they lack certainty due to questionable causality and relevance of the correlation formula for the specific country.
2. Although depending on the aspect, data are often available and relatively easy to collect.
3. Country-level data are not at all distinctive for a specific company or product. Although the Feschet and Boccoum models are based on the added value of a specific activity, the applied country data assume generically applicable income-health relations, which they are not.
4. The resulting data from empirical models are suitable for aggregation within the supply chain for the one aspect, e.g. weighted by workhours, but not between aspects.
5. Using the empirical relation between corruption and life expectancy (as health indicator) shown in Fig. 1 and Fig. 2 for corruption would be very insensitive, because at low index values it shows an almost vertical line and at high index values an almost horizontal line. In addition, for our purpose we would need a monetary midpoint- or endpoint indicator.

### ELCI-database- type-II models

Quite a number of ELCI-database- type-II models have been described. Because of the scope of this article, we will only discuss three of the most quantitative models. Baumann et al. (2013) follow standard ELCA methodology transforming the inventory data of an airbag in automobiles, into DALYs. They calculate both the lost DALYs in the production stage of the airbag, and the saved DALYs during the use of them. Following the S-LCA guideline of assessing both the positive and the negative effects, they herewith express occupational health and safety aspects in the LCA endpoints of human health.

Hunkeler (2006) however, assessing the social impact of two different detergents and considering employment a key factor to social wellbeing, proposes breaking up the life cycle inventory into labor hours, divided over different countries. Thereafter the hours are comparatively weighted by the relative labor intensity factor in the different countries or regions, and thereafter characterized by the number of workhours a person needs, to locally access a combination of societal needs. This way an assessment is made of where a company’s activity would have the most positive effect on people’s ability to fulfill their needs.

Labuschagne et al. (2006) developed a model of four social Areas of Protection, leading via a quite comprehensive list of midpoint categories to concrete characterization factors. They distinguish both quantitative and qualitative categories. Results for most categories are quantitative and made aggregable by normalizing the indicators against regional conditions and weighting by comparison with stakeholders’ perceived- and target conditions. Their quantitative indicators are concrete (e.g. number of accidents and the number of employment opportunities) and based on data availability, but therefore not necessarily the most relevant to assess the aspect. In a test on three projects, the authors attempted to collect data for both company and country levels, but concluded that at the time of testing the model, insufficient data were available. Normalization is achieved by the determination of the fraction of the results on the specific studied unit relative to those in a larger reference system, such as the GDP, regional practices, or sector output. This type of normalization is especially effective to assess the relative importance of different aspects, because it gives a comparison of the relative contribution of different aspects in a greater system (e.g. 1% of a country’s CO2 emissions and 5% of the country’s child labor), which is lost in aggregation. Normalization however often comes with an undesired weighting effect. For example, considering corruption, it will make a huge difference, if in the international arena the results are compared with the standards of a corrupt production location or with those of a conscientious consumer or supplier in a country with low corruption, in which both are stakeholders. In addition, aggregation makes little sense if used for different aspects, and not all aspects are substantively related to the reference system.

We will now assess these ELCI-database methods with our five criteria.

1. All three methods are interval quantitative. The Baumann method is quantitative and objective because data are fully based on LCIA databases and accident statistics. The data are quite certain, but only if applied under the very narrow system boundaries of only assessing direct health effects and a product that is intended to protect human health. The Hunkeler method is quantitative, objective and certain if accurate generic data are available. The Labuschagne model is not fully objective and certain because of the stakeholders’ opinion-dependent normalization and weighting.
2. Without full knowledge of the specific supply chain the labor hour data for the Hunkeler method will not be easy to collect, and the data for the Labuschagne method depend on stakeholders’ opinions, which are laborious to obtain in a relevant way and subject to fast changes. The data for the Baumann method are easier to collect because they are based on available statistics, but only if the method is applied under narrow system boundaries.
3. The Hunkeler method is not at all distinctive for a specific company or product; the Baumann case seems more distinctive, but again only under its very narrow system boundaries. Both systems could become product-distinctive with full knowledge of the specific supply chain. The Labuschagne model was meant to be distinctive for a specific company or product, but at the time not feasible because of lack of the specific data.
4. The Baumann method is suitable for aggregation within the supply chain and with other categories characterized by DALYs; the Hunkeler method is only suitable for aggregation of work-related aspects, but not between different aspects; the Labuschagne model was designed to aggregate different categories by its normalization.
5. None of these methods seems applicable to corruption; the Hunkeler method because corruption affects the whole society, not just the workers, the Baumann method because to date not enough data are available to determine corruption-related DALYs, and the Labuschagne model because of limited data availability.

Summarizing the above, quantitative impact-based S-LCA remains unsatisfactory for our goal of quantification of the aspect of corruption. In addition, the above described Tunisian incident demonstrates that if people become desperate, the consequences are totally unpredictable and that there are social tipping points, the exceeding of which may result in consequences never to be grasped in any statistical analysis. However, there are two developments towards company- and prevention-based assessment, the “Dreyer – company assessment” and the Oiconomy system.

### Dreyer—company assessment

Dreyer et al. (2006, p. 96) and Dreyer et al. (2010) argue that contrary to the assessment of environmental aspects, where the impact can be directly derived from the physical flows and processes in the life cycle of a product, social impacts are generally determined by the conduct of the companies which are engaged in the life cycle and the required specific data can only be obtained from the companies themselves. To accomplish this, a method is required both for the assessment of the companies and for the allocation of the companies’ scores to the product. Inspired by common certification standards, the authors argue that company performance can better be assessed by their efforts to prevent impact than by the impact itself, and propose an assessment method of the risk that a company presents, based on a combination of the company’s own governance quality and its contextual circumstances. For the company assessment the authors developed an extensive checklist and scoring system of “managerial measures” on three necessary “efforts”: planning, communication and active establishment and control. The three efforts are each scored one of three degrees of implementation for all managerial measures, added up and converted to a score between 0 and 1 by comparison with the maximum possible score and thereafter converted into one company risk factor by means of a complex system of division scores into risk classes and multiplication of the three effort scores, for which we refer to Dreyer et al. (2010).

A strong aspect, in our opinion, is the division in efforts and multiplicative effect of these three efforts, because non-compliance with any of the three efforts, means that governance is not effective.

However, comparing their managerial measures with the criteria in risk-based certification standards, the authors only use part of these, and choose to mix these general governance types of criteria with concrete subject-related managerial measures. In contrast, the standards leave the subject-related measures to the company, including the obligation to define these in one of the criteria, but apply, instead of three efforts, a system of more than 60 general governance criteria. It must be noted that Dreyer et al. describe a system for labor rights, where most subject-related requirements are well defined by ILO conventions. For other aspects, like safety and corruption, managerial measures may be far more company-specific, therefore less easily pre-definable and therefore better left to the company. Because of their choices Dreyer et al., in our opinion, miss the opportunity to measure the degree of company performance by means of checking and ticking the complete set of criteria and therefore apply a complex system of scoring which turns a potentially interval quantitative score into a nominal quantitative score.

After the determination of a company score, according to Dreyer et al. (2010), a share factor is required to determine the relevance of the company score for the product and, if more than one product is produced, to distribute the company impact over the different products. In principle, there are various ways to determine a share factor, but, without a standardized and verified system it will be very difficult to consistently execute such calculation over all supply chain actors. Where Hunkeler (2006), Norris (2006) and, Andrews et al. (2009) usually use workhours as the activity factor to determine the contribution or distribution of an impact in the supply chain, Dreyer et al. (2006, p.90) and Hauschild et al. (2008, p.23) argue that there is no single objective choice of an activity factor, and mention physical weight, cost contribution, value contribution and workhours as options for the distribution of a company score to a product. The disadvantage of any choice is however the same, which is the limited applicability to aspects and share- or activity factors that are relevant to each other (Hauschild et al. 2008). For example, one cannot use workhour distribution for the allocation of scores of the bribery type of corruption, because bribery negatively affects the society and competitors more than the workers.

We assess this method on our five criteria:

1. The system is nominal quantitative, objective and certain (if the data are verified).
2. Reliable data are in principle available for investigated (foreground) companies, but very difficult to obtain from background supply chain players without a certification system.
3. Part of the system is company-specific. However, the use of the generic contextual risk factor reduces its specificity.
4. If applied over a larger system in the supply chain the data are in principle technically aggregable for all aspects which are technically best quantified by the quality of the company’s governance system, and even for several aspects together, but because the result is expressed in a one risk factor value between 0 and 1, by aggregation it would totally lose its meaning. The data are not aggregable with interval quantitative data on other aspects.
5. Being based on governance principles, the system is suitable for the aspect of corruption, which the authors also mention themselves (Dreyer et al. 2010, p.258). Making this assessment however, it must be noted that the method was intended for risk assessment, not for measuring the actual status of an aspect. In order to give an overview and comparison of our assessment of the different S-LCA methods, a summary is presented in Table 1. However, we stress that this is a simplification, not fully valuing all aspects of the assessed articles.

**Table 1:** Assessment summary of SLCA methodology

	Criterion	1. Indicator quality.			2. Data		3. Distinctiveness	4. Aggregability		5. Suitability	Authors/articles involved
	Subcriterion Method	Objectivity	Certainty	Type	Background (b)/Foreground (f)	Availability		Within aspect category	Between categories	For Corruption Assessment	
Type I Checklist and scoring methods	Researchers’ based country level assessments on aspects/midpoint	Strong for generic data	Strong for generic data	Nominal	b	Strong for generic data	Weak	Strong	Weak	Strong generic data	[1,2]
	Survey based country level assesssment on aspects/midpoints	Based on stakeholders’ perceptions	Low for stakeholder obtained data	Nominal + interval	f + b	Survey based; laborious	Moderate	Strong	Weak	Weak	[2,3,4]
	LCAA	Strong for generic- or company data	Strong for company data, low for generic data	Interval	f + b	Depending on case	Depending on case and data requirements	Strong	Weak	Moderate	[5]
	SHDB	Strong for generic data	Strong for generic data	Interval	b	Global data set	Weak	Strong	Weak	Strong for generic data	[6]
Type II methods	Type II Empirical model	Strong	Weak	Interval	b	Strong	Weak	Strong	Weak	Weak	[,7,8,9,10,11,12,13]
	Type II ELCI-database model	Conditional	Conditional	Interval	f + b	Only under narrow system boundaries	Conditional	Strong	Weak	Weak	[14,15,16]
	Dreyer’s Company Assessment	Potentially strong	Potentially strong	Nominal	f	Conditional	Moderate	Weak	Weak	Strong	[17,18]

[1] = Franze and Ciroth (2011); [2] = Ekener-Petersen and Finnveden (2013); [3] = Manik et al. (2013); [4] = Foolmaun and Ramjeeawon (2013); [5] = Norris (2006) and Andrews et al. (2009); [6] = Benoît Norris et al. (2010); Benoît Norris et al. (2012); [7] = Norris (2006); [8] = Feschet et al. (2013); [9] = Hutchins and Sutherland (2008); 10 = Wu et al. (2015); 11 = Jørgensen et al. 2010b; 12 = Jørgensen et al. (2010a); [13] = Weidema (2006); [14] = Baumann et al. (2013); [15] = Hunkeler (2006); [16] = Labuschagne et al. (2006); [17] = Dreyer et al. (2006); [18] = Dreyer et al. (2010)

### Oiconomy system

Contrary to the conventional impact-based LCA, in preventative cost-based LCA, the impact is not the (un)sustainability measure, but the costs of preventing the impact. Although knowledge of the impact pathway is required to develop a preventative measure, the costs of the measure are independent of impact pathways and the perception of the seriousness. The EcoCost system (Vogtländer and Bijma 2000) and the envisioned Oiconomy system (Croes and Vermeulen 2015) weight aspects by the preventative cost-based distance to a target, or performance reference point (PRP). The EcoCost system is a conventional LCA system, intended for comparative assessment and limited to environmental aspects. It uses predetermined *marginal* preventative costs, which are the costs of the last (most expensive) employed available preventative measure necessary to reach the target (assuming that the cheapest measures are employed first). The Oiconomy system follows the supply chain, copying the financial gradual price build-up and bookkeeping in the supply chain for the hidden preventative costs of complete products. Central in the Oiconomy system is a comprehensive measuring standard (Croes 2013), leading the supply chain actor through all sustainability aspects and resulting in “Eco Social Cost Units (ESCUs)” for every aspect, added together to a total ESCU score which is transferred to the next actor in the supply chain. Every ESCU is, just as for standard financial costs, the product of a quantitative factor, provided by the supply chain actor, and a price factor. The system challenges companies to self-calculate their specific product preventative costs and only to use the predetermined marginal costs as default values where companies cannot demonstrate more accurate data. Because the Oiconomy system, by means of certification, makes all background data into foreground data, it overcomes the difficulties of the collection of product and company-specific data in S-LCA and also overcomes the need for activity variables like workhours (discussed in the section on ELCI-database- type-II models) and extends the EcoCosts ELCA-system with social aspects.

The Oiconomy system is normative and, added to the market price, aims to provide the real price of a damage-free alternative of the product. The ESCUs may be used both for comparative analyses and as a score for the sustainability of an individual product.

In order to include our new proposals for quantification of corruption in the five-criteria assessment of the Oiconomy system, we will now first present these proposals.

## Results—proposal for quantification of corruption in the Oiconomy system

We follow the standard five-step Oiconomy procedure for the determination of the marginal preventative costs (Croes and Vermeulen 2015), shortly described in the section on methods.

### Impact category and characterization factor (step 1)

The impact category is product-related corruption, characterized by the costs required to prevent corruption.

### Target, or performance reference point (step 2)

**Fig. 3:**
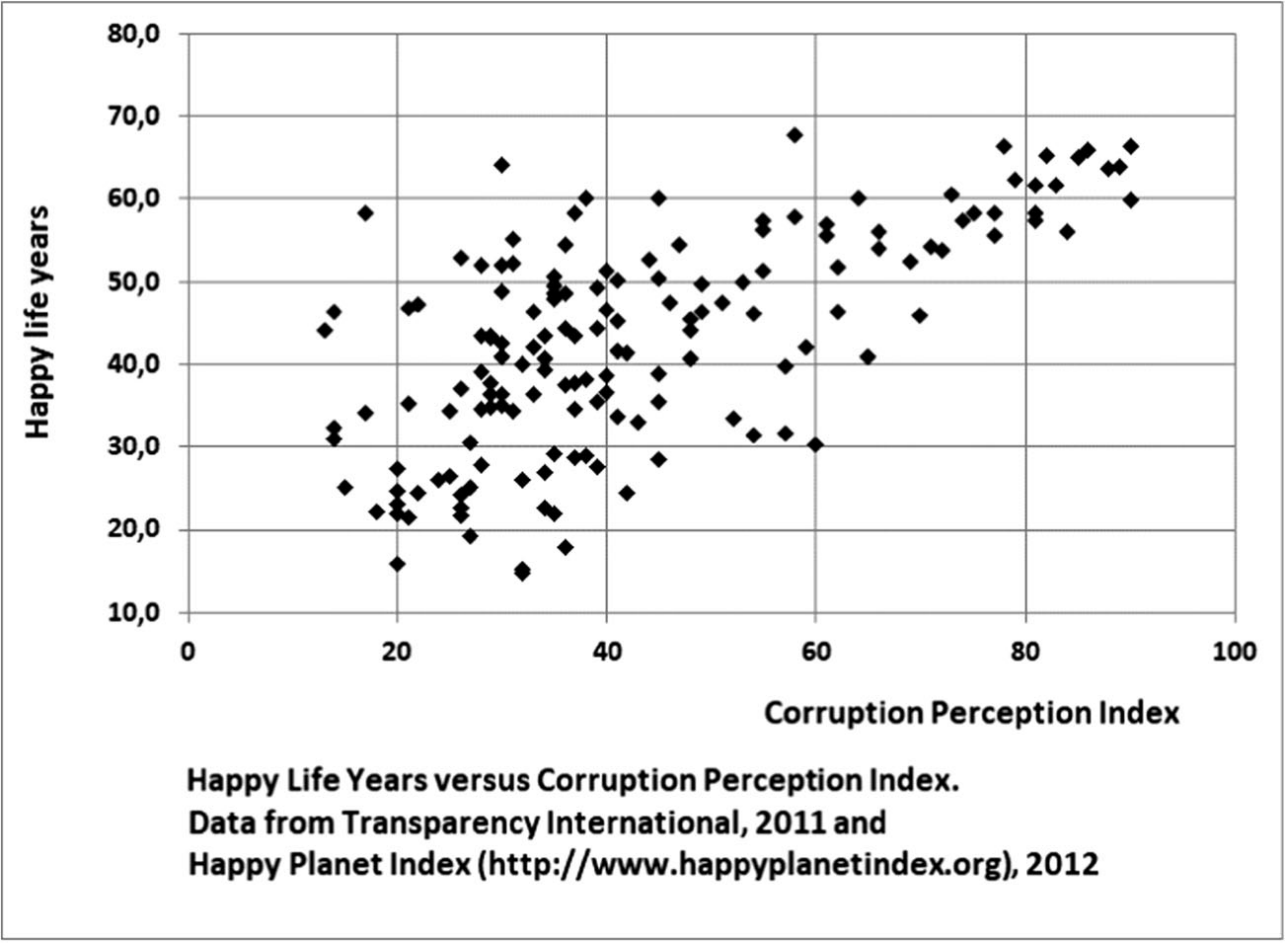
Happy Life Years versus Corruption Perception Index. Data from Transparency International 2011 and Happy Planet Index (http://ww.happyplanetindex.org), 2012

Because corruption is defined as a crime by the 2004 UN Convention against Corruption and by most countries’ legislation, the legal target or PRP must be zero corruption. However, no country is without corruption and Fig. 2 demonstrates that an index score of 43 in the Corruption Index presents a quite sharp index score above which corruption does not seem (on average) to have a severe impact on life expectancy (as indicator for health). However, if we, equally justifiably, replace life expectancy with the happy life years’ index (Fig. 3), which multiplies life expectancy with an indicator on life satisfaction, this point becomes less sharp and shifts to higher values, demonstrating that we may not exclude a corruption-induced impact on wellbeing below an index score of 64, which is only accomplished in a very limited number of countries. We therefore propose a zero target for the company but a calculation and verification tolerance in countries with a score > 64, based on data from the year 2011.

### Literature study of available preventative measures (step 3)

Palmier (1985, p. 271) identified three important causes for corruption: Low salaries, opportunities and low risk of detection and punishment, factors which according to Palmier should be addressed all three together. Quah (2011, p. 17–20) points to the cause that in many countries, civil servants’ salaries are by far insufficient to sustain their families and often lower than in the private sector. Governmental bureaucracy and regulations on the access to resources and activities provide opportunities, and if police and other members of the juridical system are also underpaid, policing against corruption will be inadequate. Important factors in prevention are transparency, political will and the example of the leadership. With corrupt leaders and policing officers, the chances of being detected and punished are perceived to be small. Although governmental bureaucracy and public officers commonly provide the demand side of corruption and business see themselves as victims (Runde et al. 2014, p. 5), business usually does provide the supply side of corruption (Myint 2000, p. 42). In addition, corruption between private companies, with businesses on both the demand and supply side, is perceived to be just as common (Heinrich and Hardoon 2011, p.19)**.**

Because corruption is an illicit activity, it is much easier to measure an organization’s preventative measures against corruption than its actual involvement in corruption incidents. There is quite a body of literature on combatting corruption e.g. (Asian Development Bank 2010; International Chamber of Commerce et al. 2008; OECD 2011; Runde et al. 2014; United Nations Global Compact 2010; World Bank 1997, 2008; Transparency International 2009; Tanzi 1998; Walcher et al. 2013; World Economic Forum 2005). All this literature demonstrates that good governance principles are key to combatting corruption. Well-developed governance systems and standards on various subjects are available, suitable for independent verification, e.g. (ISO 2004; OSHAS 2007). However, the costs of establishing and maintaining good governance are difficult to estimate, and are dependent on factors such as location and culture, availability of qualified people, size, sector, complexity, capital- and labor intensity, and experience with other operational standards.

By its business principles for countering bribery, Transparency International (2009) (TI) provides a comprehensive standard on organizational governance. The World Bank distinguishes three levels of combatting corruption for an organization. 1. Internal: Assessment of the internal risk, implementation of anti-corruption measures as part of an overall compliance program, and provision of guidance to the managers within the organization. 2. External: Sharing the internal policies with stakeholders. 3. Collective: Reaching out externally via independent facilitators to joint activities to fight corruption (World Bank 2008). Based on these principles, United Nations Global Compact (2010) provides comprehensive guidance in anti-corruption practices approaching the different stakeholders with corruption challenges. But Sedex and Verité (2013, p. 9) recommend to use the principles of common risk based management systems, such as ISO 14001, to address ethical aspects.

Because of dependence on the location, the size of the organization and specific activity, the industry sector, the stakeholders and internal organization, on collective cooperation, and the highly multi-aspect character of combatting corruption, the direct costs of measures against corruption will be very different to determine. The magnitude and persistence of bribes often depend on the ease with which the organization could relocate its activities to a less corrupt environment, which is one of the major reasons why location-dependent activities such as mining and forestry, are corruption-sensitive sectors (Bai et al. 2015; Svensson 2003, p.211; World Bank 1997, p.18). However, this also means that the ultimate measure against corruption is to refuse to pay bribes and accept the potential necessity to refrain from the activity altogether, or relocate activities away from the corrupt environment.

### Determination of the globally marginal costs preventing corruption (steps 4 and 5)

To obtain the marginal preventative costs, we need to sort the available preventative measures by their costs and deploy them starting from the cheapest until the target is reached. The last deployed measure presents the marginal preventative costs. From our literature study of currently proposed preventative measures against corruption, we recognize four cost levels.

1. Good governance costs, consisting of the costs of internal, external and collective actions.
2. Verified good governance costs, comprising governance costs, compliance to defined criteria, and external audits, which together we will call “compliance costs”.
3. Costs of refusing to pay bribes and taking the ultimate consequence of refraining from the activities.
4. Costs of actual relocation or developing alternatives for products or resources.

Considering the first level of costs, no direct data on costs of compliance to corruption criteria could be found in literature. However, the Ponemon Institute (2011, p. 13) studied compliance costs of USA industry to USA legislation, such as on privacy, data protection and regulations. The report finds a range of yearly per capita compliance costs of $ 134–$ 535, inversely related to the size of the companies. At an average yearly labor cost of about $ 60,000 per capita, that would represent about 0.22–0.89% of the wages. Considering the second cost level, more studies have been made on costs of compliance to taxation. Allers (1994) reports, based on a 1989 survey, an average compliance costs of 3% of the wage sum in the Netherlands (including costs of the tax office itself). The European Commission (2004, p.4) reports compliance costs of 0.02% of sales for large companies and of 2.6% for small and medium sized companies. These costs are very inaccurate, but this is irrelevant for our purpose, because they are lower than the costs of the marginal measure necessary to globally reduce corruption to the target level. The third level of costs for refusing to pay bribes and if necessary take the consequence of refraining from or relocating the activity, needs to be deployed. In fact, these consequences are already widely taken by all the companies that previously decided not to enter, or left corrupt markets. Fourth level costs will only be an option if these are lower than the profit margin. Therefore, we argue that, because in the long term companies will always end their activities when no profits are made, the margin made on a product represents the marginal preventative costs for the aspect of corruption. However, profit margins are volatile and may be unequally shared by companies in the supply chain, e.g. by the use of subcontractors, and multi-year product-specific averages are unusable in the case of fast moving products. Therefore, we propose to use the local five-year sector average net profit margin as a maximum default value but, without public availability of reliable local sector averages, to take the readily available S&P 500 data. An indication of the magnitude is presented in Table 2 showing the five-year sector average net profit margins in the S&P 500, from April 2016. If a company can demonstrate reliable data on all its products’ profit margins, product distinctiveness could be further improved by using the product’s share in companies’ profit as a share factor.

**Table 2:** 5-year sector average net profit margins in the S&P 500

Sector	Net profit margins (%)
Basic materials	16.47
Consumer goods	10.48
Financials	34.03
Health care	8.71
Industrial goods	6.89
Services	10.48
Technology	9.43
Utilities	20.08

(www.reuters.com Reuters 2016, accessed april 22, 2016)

The 10-year average net profit margin in the S&P 500 from 2004 to 2014 was 8.4% (Butters 2014, p. 22)

The question left now is how to derive specific company- and product ESCUs from this maximum value. Literature demonstrates that, before taking the ultimate measure of exiting or relocating, good governance is the way to combat corruption.

As described above, Dreyer et al. propose a system of scoring the degree of compliance to a set of managerial measures for the determination of a company risk factor. However, they do not use the complete set of general governance criteria commonly used in risk-based certification standards, and thereby, in our opinion and because they do not base their system on actual certification, miss the opportunity to interval quantify the effort scores. Weighting profitability against preventative measures is typically a top management responsibility. Therefore, it seems fair to set the actual preventative costs at a proportion of the measured quality of management preventative efforts. Therefore, for the Oiconomy system, we propose a modification of the Dreyer system as follows:

1. 63 common standards’ general governance criteria of equal weight are divided into four effort classes:
  - Plan (11 criteria, covering policy, risk analysis, legal requirement analysis, goals, resources).
    One of the criteria is determination of all required subject-related managerial risks and managerial measures to which all other criteria apply. The Oiconomy standard lists a number of obligatory aspects to be addressed, based on the three levels of internal, external and collective actions (World Bank 2008) and the guidance of United Nations Global Compact (2010).
  - Do (32 criteria, covering knowledge and training, communication, documentation, document control; operational control)
  - Check (16 criteria, covering emergency procedures, monitoring, evaluation, corrective measures, registration, internal audit)
  - Act (4 criteria: management review)
2. The company governance is assessed, yes or no, ticking all criteria and considering all relevant risks and managerial measures, and the scores are determined for the four effort classes. The company must realize that for a yes ticking, the criterion must be demonstrated as under control and effective.
3. The company risk (CR) is determined by the formula: CR = (Plan score/11) × (Do score/32) × (Check score/16) × (Act score/4).
4. The company ESCU score is calculated as: ESCU = (1-CR) × 5-year average net profit.
5. In the monetary Oiconomy system ESCU allocation to a product occurs exactly copying companies’ standard cost allocation methods.

The scoring system, filled with example scores, is presented in Appendix 1 (Electronic Supplementary Material).

The first three effort classes are very similar to Dreyers’ efforts. We added the fourth effort class for three reasons. 1. In common standards, the management review probably is the most important criterion, because it demonstrates continuous management commitment, evaluation and correction and requires top management to be included in audits. 2. The multiplication with an extra effort score adds to fast reduction of the company risk score with non-conformances, which is in accordance with certification practices of rejecting a certificate already with very few non-conformances. 3. The Plan-Do-Check-Act (PDCA) principles are the core of most well recognized governance standards.

The formula gives equal weight to all 4 effort classes, because compliance of all four need is necessary for effective governance. However, within the classes the criteria carry more weight the lower the number of criteria in the effort class. This answers well to the above mentioned importance of the management review with only 4 criteria in the effort class.

An explanation is required about the Oiconomy ESCU allocation of indirect costs to companies’ different products in action 5. The Oiconomy system is a bookkeeping system for hidden preventative costs, as much as possible copying companies’ financial bookkeeping system. To enable companies to exactly use their standard bookkeeping and cost allocation methods, and to avoid special “greenwashing” allocations for S-LCA purposes, for the Oiconomy system we propose to require an exact copy of companies’ standard financial cost allocation methods. The mentioned advantages outweigh the disadvantage of a not fully harmonized allocation to products, because it only concerns indirect costs (e.g. overhead). All direct costs can be allocated directly to the product.

We do not need Dreyer’s generic contextual company risk factor, because the Oiconomy system is based on actual and verified company data. Instead, as mentioned before in step 2, in order to avoid unnecessary burdens to the supply chain actors we propose to allocate zero ESCUs in countries with a corruption index score above 64, which also means that in a risk-based certification system the aspect needs no verification in such countries.

## Discussion and conclusions

Our literature review in the introduction clearly demonstrates that corruption is one of the key social aspects, heavily impacting all three PPP impact pillars of sustainability and therefore essential to be included in S-LCA.

Our assessment demonstrates that impact-based S-LCA (IB-S-LCA) methods are not suitable for product-specific interval quantification of social aspects in general and for the aspect of corruption in particular. Various authors on S-LCA have already argued that social aspects are better assessed at company level than at product level and that company performance is better determined by its preventative governance than by impact. However, for accurate product- or company-specific quantification, standardization and onsite verification are absolute requirements. The Oiconomy system (Croes and Vermeulen 2015) answers to the limitations of IB-S-LCA by standardized building up the hidden preventative costs, expressed in “Eco Social Cost Units” (ESCUs) by the supply chain actors themselves, and verification of the trustworthiness of the reported data in a certification system. The major challenge of the Oiconomy system is however, that it depends on voluntary industry submission to certification and willingness to transparent transfer of the data. In the Oiconomy system, the measure of unsustainability, related to an aspect, is characterized by the marginal costs to prevent the issue. From the literature on combatting corruption, we could derive that the marginal preventative measure against corruption is the refusal to pay bribes and take the ultimate consequence of refraining from the business, or in other words, from making a profit involving corruption. Therefore, for preventative cost-based LCA, we propose to use the 5-year sector average net profit margin, a readily available statistic, on the product as the maximum quantitative measure for the aspect of corruption. In addition, inspired by Dreyer et al., we developed a new scoring method for the quality of a company’s governance based on ticking and scoring common criteria four PDCA effort classes in risk-based certification standards, such as ISO 14001.

We will now assess our proposed method and the envisioned Oiconomy system in general on our five criteria for quantification, and discuss the limitations, consequences and potential objections to the system.

Criterion 1: Objectivity, quantitative character and certainty. The Oiconomy system in general is interval quantitative and objective, and designed to become ever more certain, because it challenges the supply chain actors to calculate their actual product-specific preventative costs on each issue and share their (anonymized) data with the system. Representing preventative costs, ESCU’s for all different aspects are expressed in one monetary unit and do not need further normalization or weighting. The system has an inherent share factor from company- to product performance, proportionally to the products’ financial share for direct costs, and equal to companies’ standard financial allocation methods for indirect costs. Unfortunately the certainty of our proposed system is limited, because multiplication of the profit margin with our company risk factor will not exactly represent the specific companies’ preventative costs. In addition, for the inherently illicit aspect of corruption, we propose an exception to the possibility for the company to present its own specific preventative costs. Therefore, for this specific aspect the ESCU scores cannot become more certain than the proposed calculation.

Criterion 2: Data availability and -complexity. By means of certification the Oiconomy system intends to make full product life cycles to foreground systems. This way, the required data for our proposed system, consisting of companies’ profit margins and assessment of compliance to a set of criteria can in principle always be made available. However, it takes a qualified auditor for a proper assessment. The creation of the required certification system itself is more complex, but there are many examples of successful certified supply chains, e.g. HACCP (food safety principles) certified food chains. The system depends on consumer pressure and the willingness of business to submit itself to a third party certification system and a double bookkeeping, one for their standard costs and one for the ESCUs.

Criterion 3: Aggregability. In the Oiconomy system, including our proposed method for corruption, the ESCUs for the different aspect categories, all representing preventative costs, are inherently normalized to preventative costs and therefore fully aggregable, both within the supply chain and between aspect categories.

Criterion 4: Distinctiveness for specific companies and products. Using foreground data, the Oiconomy system does not need an activity variable such as workhours with the problems described in our assessment of the ELCI-database methods. The ESCU score for every sustainability aspect is the product of the quantity and the price. In our proposed method for corruption, the quantity is calculated as the product of the sector-specific profit margin and a company-specific governance level-derived risk factor, totaling to a satisfactory company definition of the resulting quantification of corruption.

Before making our assessment on criterion five, the suitability of the Oiconomy system and the method proposed here for the aspect of corruption, some more limitations, consequences of, and potential objections against our methods need to be discussed.

In principle, in the Oiconomy system aspects are characterized by the costs of a concrete preventative measure, just as the Oiconomy system requires. The marginal measure against production is not paying the bribe, but the proposed indicator is the calculated proportion of the potentially corruption-infected profit margin. However in our opinion this is sufficiently close to concrete preventative costs.

Assessing social aspects involves ethical and economic considerations. In literature on S-LCA we encountered assessments based on the supposed higher positive effect of employment on human wellbeing in low income countries than in high income countries. We are strongly opposed to such assessments, because, although employment under conditions of corruption, in conflict areas, and even under poor working conditions may provide some direct income to the community, it justifies and often finances those conditions, keeps the perpetrators in power and increases the risk on suboptimal performance and therefore on future damage. In addition, in our opinion, S-LCA should avoid comparing beneficial impacts to one stakeholder group with the negatives to another group.

Regarding aspects like corruption or conflict conditions, companies find themselves choosing between being involved in the aspect, therewith enabling, playing down or even justifying corruption and preventing real long-term development, and at least providing some direct income to the poor. Companies active in corrupt or conflict areas may use the former as an excuse and object to our proposal and often even compensate by making other beneficial contributions to the community. Another excuse may be that required resources can hardly be obtained from non-corrupt countries. But profitability remains the main driver of being involved, continuing corruption-infected business as usual is not sustainable, and there is always the choice of exiting. In addition, the Oiconomy System, just like any LCA, is a measuring method; not a compliance-requiring standard.

A second point of consideration is that profits can be manipulated by the use of non-market prices and interests between interrelated businesses. Therefore, our proposals should always be used including an assessment of such manipulations, including all types of money transfers between interrelated businesses. Because certification processes are usually executed on juridical units, we stress that for a proper assessment of corruption all juridical units with related major ownerships, to or from which money transfers are made, should be included in the assessment. This can be done by the external auditor by requiring audits at the related juridical unit when there are any doubts about money transfers.

Another objection could be that, following the reasoning of this paper, the profit margin presents the costs of the marginal preventative measure for all sustainability aspects, and even for the aggregated total. Therefore, it would imply double counting. But profit is not an absolute value; it varies with the product’s prices. The costs of prevention should really be spent on prevention, and the product’s prices and margins adapted to the new costs.

A last objection could be that companies may try to hide their corruption-related activities even from the auditors. However, it is not only these direct activities that are measured, but the complete anti-corruption organization, communication, consistency, transparency and continuous improvement in the company (see Appendix 1, Electronic Supplementary Material). If there is one weakness in current certification, it is the fact that audits are usually announced. Therefore, we advise that applicants to the system should accept the possibility of unannounced audits.

Concluding in Criterion 5: Suitability for quantifying corruption. In our opinion our proposed method of applying a measured governance quality-dependent factor (calculated by the model in Appendix 1, Electronic Supplementary Material) to a maximum default value, equal to the marginal preventative costs, is not perfect, but probably the best possible for the Oiconomy system to assess social aspects that are best prevented by good governance, such as corruption, health and safety aspects, and labor conditions. Our proposed method stands the test for all but one of our five criteria: certainty. In a situation of poor governance against corruption in a high risk context, or if all managerial measures prove in vain, the indicator is certain because in that case the company should exit and lose its profit margin, if only because other companies that consciously did not enter the market, refrained from that profit. However, our proposed calculation of a governance quality-dependent fraction of that profit margin undoubtedly does not represent an exact relation between preventative costs and governance quality. However, it rewards companies’ efforts towards good governance, but also, by the multiplication effect of the four effort class scores, answers to common certification practice of refusing a certificate with even a few non-conformances.

Some words need to be said on the applicability and validity of our proposed method for the quantification of corruption in LCA. At the time of publication of this article, the Oiconomy system is in development. Sharing data with the operational EcoCost system, it is in principle suitable to become operational on environmental aspects. However, distinguishing itself by its foreground character, the Oiconomy system especially opens opportunities to include social and economic aspects to LCA. In earlier articles, we have already proposed methods for the social aspects of fair wages, fair trade, child labor and inequality (Croes and Vermeulen 2016a, b). This article is an important further step, not only because it covers the aspect of corruption, but also because we introduced a method more generally applicable to aspects that are better measured by a company’s preventative governance quality than by impact or concrete performance data.

In addition, our proposed method for the aspect of corruption is already applicable to the foreground part of any S-LCA. Based on the method, supply chain managers could have suppliers assessed, scored on corruption, and optimize their procurement based on corruption. However, the system is not applicable for background data-based LCA-systems, because it really needs onsite assessment to determine the quality of governance.

## Electronic supplementary material

ESM 1 (DOCX 23.6 kb)

## References

[CR1] Allers MA (1994) Administrative and compliance costs of taxation and public transfers in the Netherlands. Groningen University

[CR2] Andrews E (2009). Life cycle attribute assessment: case study of Quebec greenhouse tomatoes. J Ind Ecol.

[CR3] Asian Development Bank (2010) Anticorruption and Integrity. Manila

[CR4] Azfar O, Gurgur T (2008). Does corruption affect health outcomes in the Philippines?. Econ Gov.

[CR5] Bai J et al (2015) Does economic growth reduce corruption? Theory and evidence from Vietnam

[CR6] Barkemeyer R (2014). What happened to the “development” in sustainable development? Business guidelines two decades after Brundtland. Sustain Dev.

[CR7] Baumann H (2013). Does the production of an airbag injure more people than the airbag saves in traffic? Opting for an Empirically Based Approach to Social Life Cycle Assessment. J Ind Ecol.

[CR8] BBC News (2011) Mohamed Bouazizi: memories of a Tunisian martyr. January 22, 2011

[CR9] Benoît Norris C (2012) Social life cycle assessment: a technique providing a new wealth of information to inform sustainability-related decision making. In: Life cycle assessment handbook: a guide for environmentally sustainable products. Wiley, pp 433–451

[CR10] Benoît Norris C, Aulisio D, Norris GA (2010) Studying the social hotspots of 100 product categories with the social hotspots database Catherine Benoît, York, Maine

[CR11] Benoît Norris C, Aulisio D, Norris GA (2012). Identifying social impacts in product supply chains: overview and application of the social hotspot database. Sustainability.

[CR12] Benoît C, Vickery-Niederman G (2011) Social sustainability assessment literature review. Sustainability Consortium White paper

[CR13] Benoît C (2010). The guidelines for social life cycle assessment of products: just in time!. Int J Life Cycle Assess.

[CR14] Bhargava V, Bhargava V (2006). Curing the cancer of corruption. Global issues for citizens.

[CR15] Bocoum I, Macombe C, Revéret J-P (2015). Anticipating impacts on health based changes in income inequality caused by life cycles. Int J Life Cycle Assess.

[CR16] Butters J (2014) Factset Earnings Insight - S&P 500. FactSet Research Systems Inc., Norwalk

[CR17] Campos JE, Pradhan S (2007) The many faces of corruption. Tracking vulnerabilities at the sector level, Washington DC, World Bank

[CR18] Chhipi-Shrestha GK, Hewage K, Sadiq R (2015) Socializing” sustainability: a critical review on current development status of social life cycle impact assessment method. Clean Techn Environ Policy 17(3):579–596

[CR19] Croes PR (2013) Oiconomy Standard. Available at: http://oiconomy.geo.uu.nl/. Accessed 28 Jan 2017

[CR20] Croes PR, Vermeulen WJV (2015). Life cycle assessment by transfer of preventive costs in the supply chain of products. A first draft of the Oiconomy system. J Clean Prod.

[CR21] Croes PR, Vermeulen WJV (2016). In search of income reference points for SLCA using a country level sustainability benchmark (part 1): fair inequality. A contribution to the Oiconomy project. Int J Life Cycle Assess.

[CR22] Croes PR, Vermeulen WJV (2016). In search of income reference points for SLCA using a country level sustainability benchmark (part 2): fair minimum wage. A contribution to the Oiconomy project. Int J Life Cycle Assess.

[CR23] Dreyer LC, Hauschild MZ, Schierbeck J (2006). A framework for social life cycle impact assessment. Int J Life Cycle Assess.

[CR24] Dreyer LC, Hauschild MZ, Schierbeck J (2010). Characterisation of social impacts in LCA: part 1: development of indicators for labour rights - supplementary material 1-4. Int J Life Cycle Assess.

[CR25] Ekener-Petersen E, Finnveden G (2013). Potential hotspots identified by social LCA—part 1: a case study of a laptop computer. Int J Life Cycle Assess.

[CR26] Elkington J (2004). Enter the triple bottom line. The triple bottom line: does it all add up?.

[CR27] European Commission (2002) The world summit on sustainable development people, planet, prosperity

[CR28] European Commission (2004) European tax survey—working paper no. 3. Taxation Papers, pp 4–5

[CR29] Factor R, Kang M (2015). Corruption and population health outcomes: an analysis of data from 133 countries using structural equation modeling. Int J Public Health.

[CR30] Fagan C (2010). The anti-corruption catalist: realising the MDGs by 2015.

[CR31] FAO (2001) Illegal activities and corruption in the Forest sector. In: The State of the World’s Forests 2001. Rome, pp 88–101

[CR32] Feschet P (2013). Social impact assessment in LCA using the Preston pathway: the case of banana industry in Cameroon. Int J Life Cycle Assess.

[CR33] Finkbeiner M (2010). Towards life cycle sustainability assessment. Sustainability.

[CR34] Foolmaun RK, Ramjeeawon T (2013). Comparative life cycle assessment and social life cycle assessment of used polyethylene terephthalate (PET) bottles in Mauritius. Int J Life Cycle Assess.

[CR35] Franze J, Ciroth A (2011). A comparison of cut roses from Ecuador and the Netherlands. Int J Life Cycle Assess.

[CR36] GRI (2013) G4-sustainability reporting guidelines. Amsterdam

[CR37] Grießhammer R et al (2006) Feasibility study: integration of social aspects into LCA, Freiburg

[CR38] Gupta J, Vegelin C (2016). Sustainable development goals and inclusive development. Int Environ Agreements.

[CR39] Gupta S, Davoodi HR, Alonso-Terme R (2002). Does corruption affect income inequality and poverty?. Econ Gov.

[CR40] Hallak J, Poisson M (2005) Ethics and corruption in education: an overview. Int J Educ Dev 1(1)

[CR41] Hanf M (2011). Corruption kills: estimating the global impact of corruption on children deaths. PLoS One.

[CR42] Hauschild MZ, Dreyer LC, Jørgensen A (2008). Assessing social impacts in a life cycle perspective—lessons learned. Manuf Technol.

[CR43] Heckelman JC, Powell B (2008) Corruption and the Institutional Environment for Growth, Boston

[CR44] Heinrich F, Hardoon D (2011) Bribe Payers Index 2011, Berlin

[CR45] Hicks N (2013) Timber Industry & Corruption: Sub-Saharan Africa, Washington DC

[CR46] Hsu C, Wang S, Hu AH (2013) Development of a New Methodology for Impact Assessment of SLCA. In 20th CIRP International Conference on Life Cycle Engineering, Singapore, 2013

[CR47] Hunkeler D (2006). Societal LCA methodology and case study. Int J Life Cycle Assess.

[CR48] Hutchins MJ, Sutherland JW (2008). An exploration of measures of social sustainability and their application to supply chain decisions. J Clean Prod.

[CR49] International Chamber of Commerce et al (2008). Clean business is good business.

[CR50] ISO (2004) ISO 14001. Environmental management systems—requirements with guidance for use, Geneva

[CR51] ISO (2006) ISO 14040. Environmental management-life cycle assessment-principles and framework, Geneva

[CR52] Jørgensen A (2008). Societal LCA. Methodologies for social life cycle assessment. Int J Life Cycle Assess.

[CR53] Jørgensen A, Finkbeiner M et al (2010a) Defining the baseline in social life cycle assessment. Int J Life Cycle Assess 15(4):376–384

[CR54] Jørgensen A, Lai LCH, Hauschild MZ (2010b) Assessing the validity of impact pathways for child labour and well-being in social life cycle assessment. Int J Life Cycle Assess 15(1):5–16

[CR55] Koyunen C, Yilmaz R (2009). The impact of corruption on deforestation: a cross-country evidence. J Dev Areas.

[CR56] Labuschagne C, Brent AC, Labuschagne C (2006). Social indicators for sustainable project and technology life cycle management in the process industry. Int J Life Cycle Assess.

[CR57] Macombe C (2013). Social life cycle assessment of biodiesel production at three levels: a literature review and development needs. J Clean Prod.

[CR58] Manik Y, Leahy J, Halog A (2013). Social life cycle assessment of palm oil biodiesel: a case study in Jambi Province of Indonesia. Int J Life Cycle Assess.

[CR59] Mauro P (1997) The effects of corruption on growth, investment, and government expenditure: a cross-country analysis. Institute for International Economics, pp 83–107

[CR60] Myint U (2000). Coruption: causes, consequences and cures. Asia-Pacific Development Journal.

[CR61] Norris GA (2006). Social impacts in product life cycles. Towards life cycle attribute assessment. Int J Life Cycle Assess.

[CR62] OECD (2011) OECD guidelines for multinational enterprises. OECD Publishing

[CR63] OECD (2013) G20 document: issues paper on corruption and economic growth. OECD Publishing

[CR64] OECD & The Council of Europe (2012) The Multilateral Information Convention on Mutual Solutions Administrative Technology Assistance in Tax Matters - Amended by the 2010 Protocol. OECD Publishing

[CR65] OSHAS (2007) OHSAS 18001 Health & Safety Standard

[CR66] Palmier L (1985). The control of bureaucratic corruption: case studies in Asia.

[CR67] Ponemon Institute (2011). The true cost of compliance a benchmark study of multinational organizations.

[CR68] Quah JST (2011) Curbing corruption in Asian countries: an imposible dream? Emerald Group Publishing

[CR69] Reuters (2016) Financial Highlights. Available at: http://www.reuters.com/finance/stocks/financialHighlights?symbol=DPS. Accessed 22 Apr 2016

[CR70] Runde D.F., Hameed, S. & Magpile, J., 2014. The costs of corruption. Strategies for Ending a Tax on Private-sector-led Growth, Washington DC

[CR71] SDSN (2015) Getting started with the sustainable development goals. A Guide or Stakeholders

[CR72] Sedex & Verité (2013) Businesss Ethics. In: Sedex Supplier Workbook. Supplier Ethical Data Exchange, London

[CR73] Søreide T (2014) Drivers of corruption: a brief review. World Bank

[CR74] Svensson J (2003) Who must pay bribes and how much? Evidence from a cross section of firms. Q J Econ 207–230

[CR75] Swarr TE (2009). Societal life cycle assessment-could you repeat the question?. Int J Life Cycle Assess.

[CR76] Tanzi V (1998). Corruption around the world. Causes, consequences, scope and cures. IMF Staff Pap.

[CR77] Transparency International (2009) Business principles for countering bribery. A Multi-Stakeholder Initiative led by Transparency International, Berlin

[CR78] Transparency International (2011) Corruption Perceptions Index 2011, Berlin

[CR79] Transparency International (2013). Global corruption report: climate change.

[CR80] Transparency International (2017) Corruption Perceptions Index 2017

[CR81] UNEP/SETAC (2009) UNEP guidelines for social life cycle assessment of products, UNEP/Earthprint

[CR82] United Nations Global Compact (2010) Fighting corruption in the supply chain: a guide for customers and suppliers. New York

[CR83] United Nations Global Compact & Transparency International (2009) Reporting guidance on the 10th principle against corruption. New York

[CR84] United Nations Office on Drugs and Crime (2004) United Nations convention against corruption, Vienna

[CR85] Vermeulen WJV, Witjes S (2016). On addressing the dual and embedded nature of business and the route towards corporate sustainability. J Clean Prod.

[CR86] Vogtländer JG, Bijma A (2000). The ‘virtual pollution prevention costs 99. A single LCA-based Indicator for emissions. Int J Life Cycle Assess.

[CR87] Vogtländer JG, Brezet HC, Hendriks CF (2000). The virtual eco-costs ’ 99. Int J Life Cycle Assess.

[CR88] Walcher A, Stempkowski R, Apfalter M (2013) Prevent Corruption – Measures to Increase Integrity in Organizations. In Life-Cycle and Sustainability of Civil Infrastructure Systems, 2013. Vienna

[CR89] Wei S-Y (1999) Corruption in economic development: beneficial grease, Minor Annoyance, or Major Obstacle? Washington DC

[CR90] Weidema BP (2006). The integration of economic and social aspects in life cycle impact assessment. Int J Life Cycle Assess.

[CR91] World Bank (1997) Helping Countries Combat Corruption: The role of the World Bank. Poverty Reduction and Economic Management, (September)

[CR92] World Bank (2008) Fighting corruption through collective action, Washington DC

[CR93] World Bank (2016) Empowerment - empowering the poor to fight corruption. World Bank website. Available at: http://web.worldbank.org. Accessed 2 Mar 2016

[CR94] World Economic Forum (2005) Partnering Against Corruption - Principles for Countering Bribery, Geneva

[CR95] Wu SR (2015). Causality in social life cycle impact assessment (SLCIA). Int J Life Cycle Assess.

