# Supplementary material for: Quantification of corruption in preventative cost-based S-LCA: a contribution to the Oiconomy project
Source: Int J Life Cycle Assess. 2018 Sep 6;24(1):142–59. doi: 10.1007/s11367-018-1507-z (PMC6383569; doi:10.1007/s11367-018-1507-z)
Supplement: Supplementary file 1 — (DOCX 23.6 kb) [file 11367_2018_1507_MOESM1_ESM.docx]

Electronic Supplementary Material

societal lca

**Quantification of corruption in preventative costs based S-LCA. A contribution to the Oiconomy Project**

**Pim R. Croes^1^ • Walter J.V. Vermeulen^1^**

^1^Utrecht University – Copernicus Institute of Sustainable Development, Utrecht, Heidelberglaan 2, 3584 CS Utrecht, The Netherlands

Received: 21 August 2016 / Accepted: 11 July 2018

© Springer-Verlag GmbH Germany, part of Springer Nature 2018

Responsible editor: Marzia Traverso

🖂 Pim R. Croes

p.r.croes@uu.nl

**Appendix 1**  Corruption Governance Level Scoring Model

| Filled criteria-scores (0 or 1) are an example. The scores of the PDCA Effort Categories are calculated from the criteria scores.  All criteria to be assessed considering the aspect of corruption. | |  |
| --- | --- | --- |
| **PDCA Class and nr.** | **Criterion (demonstrated and effective)** | **Scores** |
|  |  |  |
|  | Plan Category = Sum Plan-criteria **/** Number Plan-criteria | 0.91 |
|  | Do Category = Sum Do-criteria **/** Number Do-criteria | 0.59 |
|  | Check Category = Sum Check-criteria **/** Number Check-criteria | 0.88 |
|  | Act Category = Sum Act-criteria **/** Number Act-criteria | 0.75 |
|  | **Company Score = Plan x Do x Check x Act** | 0.35 |
|  | **Calculation factor to be multiplied with maximum ESCU score (= 1- company score)** | **0.65** |
|  |  |  |
| **Plan** | **Policy.** | **criteria scores (0 or 1)** |
| 1.1 | Policy defined, appropriate and up-to-date, and approved by the highest local management and by the highest corporate management. | 1 |
| 1.2 | Policy is aimed at continuous improvement. | 1 |
| 1.3 | Policy includes compliance to the national law and to the requirements of the Oiconomy Standard. | 1 |
| 1.4 | Documented, maintained and implemented plan of goals in a (at least five years) plan. Decision structure and Tasks, Responsibilities (T.C.R.s) defined. | 1 |
| **Plan** | **Risk analysis** |  |
| 2.1 | Procedure for a risk analysis. | 1 |
| 2.2 | Analysis of the relevant risks for all stakeholders. | 1 |
| 2.2 | All risks and measures listed in the relevant section of the Oiconomy Standard included in the analysis. | 1 |
| 2.3 | Risks are ranked and priorities are set based on the seriousness and frequency (or ease) of occurrence. | 0 |
| **Plan** | **Legal requirements** |  |
| 3.1 | Analysis of the relevant legal requirements and international declarations and for which activities related to the product these requirements are relevant. | 1 |
| **Plan** | **Goals** |  |
| 4.1 | Conversion of the policy goals into SMART goals. (Specific, Measurable, Acceptable, Realistic, Time limited) | 1 |
| 4.2 | Plan of implementation and monitoring on the SMART goals. | 1 |
| **Do** | **Resources available** |  |
| 5.1 | Qualified people with defined T.C.R. s, funds, time and investments. | 0 |
| 5.2 | Qualified coordinator with access to the highest local and corporate management. | 1 |
| 5.3 | Top management commitment demonstrated by the documentation and communication. | 1 |
| 5.4 | Required capital investments determined, and approved by the highest (corporate) management, and in the planned stage of execution. | 0 |
| **Do** | **Knowledge and training** |  |
| 6.1 | Required knowledge and skills throughout the organization defined and made available. | 1 |
| 6.2 | Needs for training analyzed and training provided where necessary. | 1 |
| 6.3 | Availability of knowledge documents and, where necessary, contacts with relevant experts. | 0 |
| **Do** | **Internal and external communication** |  |
| 7.1 | The plan and T.C.R.s are communicated internally and externally. | 1 |
| 7.2 | Consciousness and relevant knowledge on the relevant aspect throughout the organization. | 1 |
| 7.3 | Analysis of all internal and external stakeholders that may have an interest or influence on the relevant aspect. | 1 |
| 7.4 | Analysis of all internal and external stakeholders that may experience an adverse effect regarding the relevant aspect. | 0 |
| 7.5 | Plan of communication with all defined stakeholders. | 1 |
| 7.6 | All risks and information are communicated to the relevant stakeholders in a clear, readily accessible, understandable, unambiguous way. The company is readily available for complaints. | 0 |
| **Do** | **Documentation** |  |
| 8.1 | Policy, scope and working of the relevant management system are documented. | 1 |
| 8.2 | Documents required by the Oiconomy Standard for ESCU registration. | 1 |
| 8.4 | Procedures that regulate the working of the system. | 1 |
| 8.4 | Methods of measuring and sampling. | 1 |
| 8.5 | Descriptions of functions, T.C.R.s and the organization structure. | 0 |
| **Do** | **Document control** |  |
| 9.1 | Authorization of documents for first use and for revision. | 1 |
| 9.2 | Documents identifiable, up-to-date, legible and readily available where needed. | 1 |
| 9.3 | Prevention of use of unauthorized documents. | 1 |
| 9.4 | Identification of required external documents and controlled distribution thereof to the relevant locations and functions. | 1 |
| **Do** | **Operational control** |  |
| 10.1 | Criteria identified based on requirements, risk analysis and goals. | 1 |
| 10.2 | System of preventative measures on identified risks. Analysis of those (critical control) points internally (and if necessary externally) where absolute prevention can be achieved. Effective prevention systems on these points. | 1 |
| 10.3 | Procedures to discover shortcomings and to convert these into improvements, including a procedure for handling complaints and incidents with a time limit for completing these, and including a procedure for protection of whistleblowers. | 1 |
| 10.4 | Control of the influence of third parties on the relevant aspect, including a procedure to monitor and evaluate the quality of the suppliers and the transparency of their communication. | 0 |
| **Do** | **Emergency plans** |  |
| 11.1 | Action plans for incidents of corruption and non-compliance. | 0 |
| 11.2 | Yearly evaluation of incidents of corruption and non-compliance. | 0 |
| 11.3 | Yearly tests of emergency situations where practical. | 0 |
| 11.4 | Traceability at least one step backwards and one step forwards, including waste flows. | 0 |
| 11.5 | Procedure of internal and external communication in case of a corruption incident, -request or -threat. | 0 |
| 11.6 | Yearly test of traceability (including waste) and communication procedure. | 0 |
| **Check** | **Monitoring and measurement on the relevant aspect.** |  |
| 12.1 | Plan and execution of monitoring (what, when, where and how), fit for demonstration that the control system is effective. Inclusion of near-incidents. | 0 |
| 12.2 | Use of independent persons for monitoring and avoidance of personal involvement of these persons. | 0 |
| **Check** | **Evaluation of the relevant aspect.** |  |
| 13.1 | Procedures for periodical evaluation of the monitoring results against the requirements and goals. | 1 |
| 13.2 | Procedures for registration and communication of the monitoring results and of the conclusions of the evaluation. | 1 |
| **Check** | **Preventive and corrective measures on the relevant aspect.** |  |
| 14.1 | Procedures to discover the root causes of shortcomings, incidents and complaints and to prevent repetition thereof. (Consider the possibility that top management may ultimately be the root cause, especially on repetition of the shortcoming). | 1 |
| 14.2 | Procedures for preventive measurements based upon the risks found in the risk analysis. | 1 |
| 14.3 | Procedures to analyze trends in incidents, shortcomings and complaints and to improve the policy. | 1 |
| 14.4 | Procedures to compensate, remove or minimize the damage that has been caused by incidents or shortcomings. | 1 |
| 14.5 | Monitoring on and registration of preventive and corrective measures. | 1 |
| 14.6 | Procedures to regularly verify and evaluate the adequacy of earlier taken preventive and corrective measures. | 1 |
| **Check** | **Registration and control.** |  |
| 15.1 | Meeting the requirements is demonstrable by registered data. | 1 |
| 15.2 | Registered data are traceable, stored in a protected way, available for at least five years, legible and identifiable. | 1 |
| 15.3 | Coherent and communicated system of registration locations. | 1 |
| **Check** | **Internal audit on the relevant aspect.** |  |
| 16.1 | At least yearly internal audit by independent (no auditing of people’s own responsibilities), objective and qualified persons against the requirements. | 1 |
| 16.2 | Written report with information to the management. | 1 |
| 16.3 | Audit procedure with criteria, scope, method, frequency, reporting, shortcomings, requirements for evaluation and corrective measures. | 1 |
| **Act** | **Management review on the relevant aspect.** |  |
| 17.1 | At least yearly management review. | 1 |
| 17.2 | Top management responsibility is demonstrable in practice. | 1 |
| 17.3 | Documented input: Internal audit, complaints, incidents, relevant communication with authorities and other stakeholders, evaluations, status of preventive and corrective measures, result of the decisions of the last MR, changes in this standard, legislation and other requirements and improvement propositions. | 0 |
| 17.4 | Documented output: assessment of the effectiveness of the system, decisions for improvement; adjustments in the policy, goals and plans. | 1 |
